# Size- and environment-driven seedling survival and growth are mediated by leaf functional traits

**DOI:** 10.1098/rspb.2022.1400

**Published:** 2022-09-28

**Authors:** Feng Jiang, Marc W. Cadotte, Guangze Jin

**Affiliations:** ^1^ Center for Ecological Research, Northeast Forestry University, Harbin 150040, People's Republic of China; ^2^ Department of Biological Sciences, University of Toronto Scarborough, Toronto, ON, Canada; ^3^ Ecology and Evolutionary Biology, University of Toronto, Toronto, ON, Canada; ^4^ Key Laboratory of Sustainable Forest Ecosystem Management–Ministry of Education, Northeast Forestry University, Harbin 150040, People's Republic of China; ^5^ Northeast Asia Biodiversity Research Center, Northeast Forestry University, Harbin 150040, People's Republic of China

**Keywords:** environmental filtering, intraspecific trait variation, forest dynamics plot, plant ontogeny, structural equation modelling

## Abstract

Ecologists usually find that plant demography (e.g. survival and growth) changes along with plant size and environmental gradients, which suggests the effects of ontogeny-related processes and abiotic filtering. However, the role of functional traits underlying the size– and environment–demography relationships is usually overlooked. By measuring individual-level leaf traits of more than 2700 seedlings in a temperate forest, we evaluated how seedling traits mediated the size– and environment–demography relationships. We found leaves were larger for taller seedlings; leaf economics traits were more conservative in taller seedlings and under high-light and low-elevation conditions. Structural equation modelling showed that a higher survival probability for taller seedlings was indirectly driven by their larger leaf area. Although taller seedlings had lower growth rates, larger and more resource-conservative leaves could promote the growth of these tall seedlings. Environmental variables did not influence seedling survival and growth directly but did influence growth indirectly by mediating trait variation. Finally, species-specific variation in traits along with size and environments was associated with the species-specific variation in seedling survival and growth. Our study suggests that not only plant ontogeny- and environment-related ecological processes, but functional traits are also important intermediary agents underlying plant size– and environment–demography relationships.

## 1. Introduction

Variations in plant demography such as survival and growth can ultimately determine species abundance, community structure and diversity, therefore, determining the mechanisms underlying plant demographic dynamics is important to understand how forest communities are assembled and change over time. The seedling stage is a bottleneck stage for plant survival and growth because of the production of more individuals than could occupy open spaces and their general susceptibility to surrounding abiotic and biotic environments [1]. Given this bottleneck, seedling demography such as survival and growth is a pivot point that is critical to understanding species coexistence and community assembly [2–5]. Recent studies have found that multiple biotic and abiotic factors can influence seedling growth and survival [4,6,7]. However, the reasons why these factors influence seedling survival and growth are not very clear.

**Figure 1.**
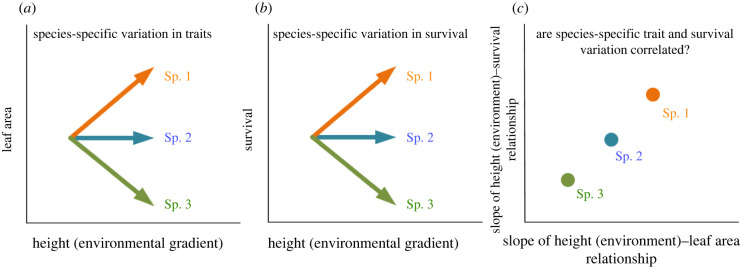
An illustration of how species-specific variation in traits is associated with species-specific variation in survival along the height and environmental gradients. (*a*) Species traits such as leaf area could increase, decrease or remain along height or environmental gradients; (*b*) if trait variation shown in (*a*) influences seedling survival, the survival of these species is expected to have specific responses along these gradients. Therefore, in (*c*), we expect that the slopes of height (environment)–trait relationships are associated with the slopes of height (environment)–survival relationships across species. These hypotheses can be applied to the seedling growth model. (Online version in colour.)

With the increasing number of studies exploring seedling survival and growth, especially in permanent forest dynamics plots, most studies find that seedling height is the most important factor influencing survival and growth, and superior to other biotic and abiotic factors (the size–demography relationship [1,4,5,8–12]). Generally, survival probability is higher, and growth is slower for taller and thus usually older seedlings. However, given the importance of height on seedling survival and growth, few studies have given a clear explanation for the mechanisms underlying this seedling size–demography relationship. One reason is that tall and old seedlings have better positions to access light or have been filtered by abiotic or biotic factors through many years to adapt to their environments. Another potential reason is that seedling functional traits change as seedlings become taller, such as increased allocation to physical defence or adopting a more resource-conservative strategy, which is against surrounding abiotic and biotic environments. More biomass allocated to defence rather than photosynthesis will slow down the growth of seedlings. However, few studies have examined this hypothesis, especially at the plant early life stages (but see [13]). In this study, we use an individual-level leaf trait dataset that included most coexisting woody species in a temperate forest to explore how seedling traits vary with increased height, and evaluate whether this height-driven trait variation influences seedling survival and growth (figure 1; electronic supplementary material, figure S1).

Regardless of the direct effect of seedling height on growth and survival, variation in abiotic conditions, such as light availability and habitat, also influence seedling survival and growth (the environment–demography relationship [4,5,14]). According to the environmental filtering hypothesis, abiotic factors can inflict stress on individual plants, which increases the likelihood of mortality and so filters out plants that cannot cope (electronic supplementary material, figure S1). This hypothesis is assumed as an important ecological process influencing community assembly [15,16]. The alternative view would be a more indirect causal pathway, where plant functional traits are influenced by environmental gradients [17,18] and that functional trait variation can influence variation in plant demographic rates [19,20]. Thus, the environment–trait relationship can also contribute to the relationships between environmental gradients and demographic rates (electronic supplementary material, figure S1, trait plasticity hypothesis). Distinguishing between these two different hypotheses can improve our understanding of the relative importance of trait turnover (intraspecific and interspecific) and environmental change on plant demographic rates along with the environmental gradients (electronic supplementary material, figure S1).

Recent studies suggest that co-existing species usually show species-specific trait variation along with increased plant size [21–23] and environmental gradients [18,24]. If functional traits influence plant demographic rates, this species-specific variation in functional traits will determine species-specific variation in plant demographic rates along with plant size and environmental gradients (figure 1*c*; [4,23]). Exploring this relationship can help us to understand how species demographic rates change along with their ontogenetic stages and environmental gradients by mediating their functional traits.

In a temperate forest in Northeast China, we measured a total of 10 individual-level lamina and petiole traits for more than 2700 seedlings to explore how seedling functional traits mediated size–demography (survival and growth) and environment–demography relationships. Here we ask the following questions: (i) how do the seedling leaf traits change along with height and environments (light availability and elevation)? (ii) Can height and environmental factors influence seedling survival and growth directly, or indirectly by changing leaf traits (electronic supplementary material, figure S1)? (iii) Can species-specific variation in leaf traits trait determine species-specific variation in their survival and growth along with height and environmental gradients (figure 1)?

## 2. Material and methods

### (a) Study site

This study was conducted in the Liangshui National Natural Reserve (47°10′50″ N, 128°53′20″ E) in Northeast China. The climate is temperate continental monsoon with most rainfall in summer. The mean annual temperature is −0.3°C and the mean annual precipitation is 676 mm. The mixed broadleaved-Korean pine (*Pinus koraiensis*) forest is the most common forest type in Northeast China, and the Liangshui Reserve preserves a large amount area of these primary and undisturbed forests. In this Reserve, we established a 9 ha (300 m × 300 m) forest dynamics plot in the undisturbed area and all woody plants with the diameter at breast height (DBH) ≥ 1 cm have been tagged, identified, mapped and measured (DBH) [25]. This plot was located on a slope with gradually increased elevation from 425 m to 508 m (electronic supplementary material, figure S2), which created a continuous environmental gradient across our plot. Our previous studies have found that elevation was an important factor that influenced tree distribution and survival in this plot [26,27]. Within the period of our study, there were no drastic climate change or extreme weather events that might influence our observation of plant survival and growth.

### (b) Seedling plots and trait collections

Within the 9 ha big plot, we established a total of 900 seedling plots of 4 m^2^ (2 m × 2 m) at the intersections of a 10 m grid (electronic supplementary material, figure S2). In these seedling plots, we censused all woody plants with height ≥ 10 cm and DBH < 1 cm. Seedling height was measured, and survival status was recorded in 2018 and then re-censused in 2020. In August 2018, we collected the leaves of all seedlings except for lianas and conifers in 283 plots that were distributed widely in the big plot (electronic supplementary material, figure S2). The definition of seedling here using plant size was not absolute for each species, but represented the relative early life stage and has been used in many forest dynamics plots [1,3]. To decrease damage to the seedlings, we sampled only one leaf for small seedlings with few leaves, or two leaves for large seedlings with many leaves at the end of the growing season. The healthy and mature leaves with petiole were sampled and placed in a foam box. Ice blocks were also placed in the box to keep the box cold and decrease the water loss from the leaves. These samples were transferred to the laboratory for leaf trait measurements within 4 h.

We measured a total of 10 leaf lamina and petiole traits and divided them into five groups of traits: lamina size traits (lamina area, LA, cm^2^; lamina thickness, LT, mm), lamina economics traits (specific lamina area, SLA, cm^2^/g; lamina dry matter content, LDMC, g/g; lamina chlorophyll content, Lchl, mass-based SPAD value), petiole size traits (petiole length, PL, cm; petiole diameter, PD, mm), petiole economics traits (specific petiole length, SPL, cm/g; petiole dry matter content, PDMC, g/g) and the whole leaf trait (lamina matter ratio, LMR, g/g). We grouped these traits because recent studies found that leaf size and economics traits were decoupled [28]. We measured LT (mm) using a micrometer (0.01 mm) and LA by scanning them. Lamina and petiole's fresh weights were measured by an analytical balance (0.0001 g) and then oven-dried at 60°C for constant weight. SLA was estimated as LA divided by lamina dry weight. LDMC was determined as the lamina dry weight divided by fresh weight. Lamina chlorophyll content per area was measured by the SPAD-502 Plus meter (Konica Minolta, Inc., Japan) and then transformed to mass-based chlorophyll content (Lchl) by multiplying SLA. PL was measured by a ruler (0.1 cm). PD (0.01 mm) was determined by a micrometer. SPL was calculated as PL divided by petiole dry weight. PDMC was generated as petiole dry weight divided by fresh weight. LMR was the lamina dry weight divided by the whole leaf dry weight. Four special cases needed to be clarified for sampled leaves and trait measurements. First, we double-identified the species name by checking the scanned lamina images and found that 11 liana seedlings (two for *Schisandra chinensis* and nine for *Actinidia kolomikta*) had been sampled, and we included these measurements in our analyses in survival and growth models (the second section). Second, some seedlings with no leaves or with only unhealthy leaves (e.g. yellowed) were not sampled. Third, if the leaves were grazed partly by herbivores (e.g. small holes), we used Photoshop CS6 to green the grazed parts to generate a more accurate estimate of LA. This corrected LA was highly correlated with the original estimates (*R*^2^ = 0.998). Fourth, for the compound leaves, we used the leaflet as the trait measurement, but for LMR, we used all leaflets in a petiole. Finally, we excluded the observations with lamina and petiole dry matter < 0.0040 g and petiole length ≤ 0.2 because of the potentially large errors when we measured them using the analytical balance and ruler. Eventually, we included a total of 2747 individuals of 33 species in 281 plots.

### (c) Light availability and elevation

We estimated the light availability in seedling plots using hemispherical photographs (Nikon Coolpix 4500 digital camera with a 180° fish-eye lens; [29]). We took one picture at each seedling plot (1 m above-ground). Light availability was obtained as the canopy openness index using the Gap Light Analyser software. The elevation was measured using the total station when we established the forest dynamics plot [27].

### (d) Statistics

#### (i) The effects of seedling height and environmental factors on leaf trait variation

We used multiple regression models to explore the effects of seedling height, light availability and elevation on each leaf trait of each species [18]. In these models, height, light and elevation were predictors, and leaf traits were the response variables. Models were fitted for each species using the *lm* function in R 4-0-0 [30]. For this analysis, we only selected species with more than six individuals and presented them in more than two plots. All functional traits and seedling height were log10-transformed for all analyses in this study.

#### (ii) Height- and environment-driven trait variation affect seedling survival and growth

To understand how seedling height, light and elevation influenced survival and growth directly and indirectly by mediating trait variation, we used a structural equation modelling framework to evaluate the relationships among seedling height, environments, leaf traits, survival and growth (electronic supplementary material, figure S1, [31]). We select LA and the lamina economics spectrum (LES) trait as two trait variables in the structural equation modelling. LES was generated from the first principal component axis of specific lamina area, lamina chlorophyll content and lamina dry matter content which were strongly correlated (electronic supplementary material, figure S3). Higher LES values represented acquisitive leaf strategy with higher specific lamina area, lamina chlorophyll content and lower lamina dry matter content. Pearson correlations showed that there were significant but weak correlations among LA, lamina thickness and LES (electronic supplementary material, figure S3). We did not include lamina thickness because it did not show relationships with seedling survival and growth and resulted in poor performance of the structural equation modelling (i.e. *p* < 0.05). Structural equation models were constructed for seedling survival and growth, respectively (electronic supplementary material, figure S1). Within the survival structural equation modelling framework, we used the generalized linear mixed model to fit the seedling survival and linear mixed models to fit leaf traits and seedling height (electronic supplementary material, figure S1). The formulas were as follows:

2.1 $$\begin{array}{rl} {\mathrm{Survival}}_{\left[i\right]}\;=\; & \beta_{0}+\beta_{1}LA_{i}+\beta_{2}LES_{i}+\beta_{3}{\mathrm{Height}}_{i}\\ & +\beta_{4}{\mathrm{Light}}_{i}+\beta_{5}{\mathrm{Elevation}}_{i}+\Phi_{q}+\gamma_{s}\\ & {\mathrm{Survival}}_{\left[i\right]}∼\mathrm{Bernoulli}(\theta_{i}) \end{array}$$

2.2 $${\mathrm{Trait}}_{\left[i\right]}=\beta_{0}+\beta_{1}{\mathrm{Height}}_{i}+\beta_{2}{\mathrm{Light}}_{i}+\beta_{3}{\mathrm{Elevation}}_{i}+\Phi_{q}+\gamma_{s}$$

2.3 $${\mathrm{Height}}_{i}=\beta_{0}+\beta_{1}{\mathrm{Light}}_{i}+\beta_{2}{\mathrm{Elevation}}_{i}+\Phi_{q}+\gamma_{s}$$

Survival_[*i*]_ is the survival status (0, dead; 1, alive) of the *i*_th_ seedling from 2018 to 2020; trait_[*i*]_ is the trait value of the *i*_th_ seedling. *Φ*_q_ and *γ*_s_ were random effects of quadrat and species.

Within the growth structural equation modelling framework, we used linear mixed models to fit growth rates (equation (2.4)), trait values (equation (2.2)) and height (equation (2.3)), respectively. The formula is as follows:

2.4 $$\begin{array}{rl} {\mathrm{Growth}}_{\left[i\right]} & =\beta_{0}+\beta_{1}LA_{i}+\beta_{2}LES_{i}+\beta_{3}{\mathrm{Height}}_{i}+\beta_{4}{\mathrm{Light}}_{i}\\ & \;+\beta_{5}{\mathrm{Elevation}}_{i}+\Phi_{q}+\gamma_{s} \end{array}$$

Growth_[*i]*_ is the relative growth rate of the *i*_th_ seedling. Seedling relative growth rate was calculated as (ln(height_t1_)–ln(height_t0_))/2 [32], where height_t0_ and height_t1_ were seedling heights in 2018 and 2020, respectively, and 2 was a 2-year growth period. Equations (2.2) was modelled for LA and LES, separately. For the growth rate, we excluded observations of negative values (461 seedlings) and values larger than 95% quantile (84 seedlings) [32]. Our main results did not change by including the negative growth values (results not shown). Finally, there were a total of 2632 individuals and 33 species in 278 seedling plots in the survival model and a total of 998 individuals and 33 species in 248 plots in the growth model. Generalized linear mixed models and linear mixed models were performed using the *glmer* and *lmer* functions in the lme4 package [33]. Structural equation models were calculated using the *psem* function in the piecewiseSEM package [34]. Environmental factors were standardized using the *scale* function, and functional traits and height were log10-transformed. We used standardized coefficients to describe the relationships in the structural equation modelling.

#### (iii) Relationships between species-specific trait variation and demographic rates

We used two-level mixed models to evaluate whether species-specific variation in traits could explain the species-specific variation in survival and growth along with increased height and environmental gradients (figure 1*c*). In this section, we evaluated five lamina traits: LA, LT, SLA, Lchl and LDMC with a large number of samples. Species with more than six individuals and presented in more than two seedling plots were included in the mixed models. The first-level models are as follows:

2.5 $$\begin{array}{rl} {\mathrm{Survival}}_{\left[i\right]}=\; & \beta_{0}+\beta_{1s}{\mathrm{Height}}_{i}+\beta_{2s}{\mathrm{Light}}_{i}\\ & +\beta_{3s}{\mathrm{Elevation}}_{i}+\Phi_{q}+\gamma_{s}\\ & {\mathrm{Survival}}_{\left[i\right]}∼\mathrm{Bernoulli}(\theta_{i}) \end{array}$$

2.6 $${\mathrm{Growth}}_{\left[i\right]}=\beta_{0}+\beta_{1s}{\mathrm{Height}}_{i}+\beta_{2s}{\mathrm{Light}}_{i}+\beta_{3s}{\mathrm{Elevation}}_{i}+\Phi_{q}+\gamma_{s}$$

2.7 $${\mathrm{Trait}}_{\left[i\right]}=\beta_{0}+\beta_{1s}{\mathrm{Height}}_{i}+\beta_{2s}{\mathrm{Light}}_{i}+\beta_{3s}{\mathrm{Elevation}}_{i}+\Phi_{q}+\gamma_{s}\;$$

From equations (2.5)–(2.7), we allowed the effects of height, light and elevation to vary among species (i.e. *β_1 s_*, *β_2 s_*, and *β_3 s_*; species-specific slopes shown in figure 1*a,b*). The second-level models were modelled using linear models where we used species-specific *β_1_*, *β_2_* and *β_3_* that were extracted in the trait models (equation (2.7)) to predict species-specific *β_1_*, *β_2_* and *β_3_* that were extracted in the survival (equation (2.5)) and growth (equation (2.6)) models, respectively (as shown in figure 1*c*). Therefore, equations (2.5)–(2.7) are different from equations (2.1)–(2.4) because the former models included species as a random slope and were not performed within a structural equation modelling framework. To control the sample size, the dataset used in trait models was the same as the survival and growth models, respectively. Height was log10-transformed and all predictors were standardized.

## 3. Results

### (a) Leaf trait variation along with height, light and elevation

**Figure 2.**
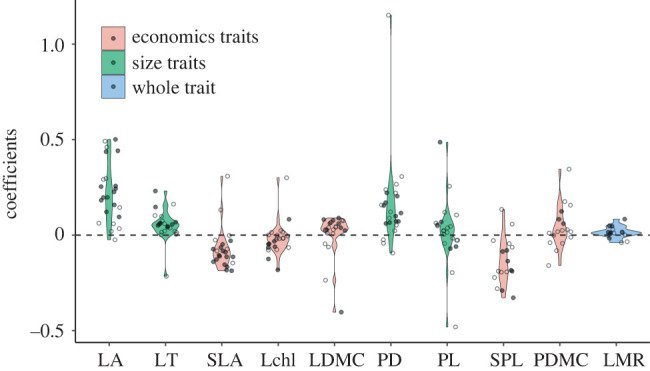
Effects of seedling height on leaf trait variation (i.e. coefficients) in the multiple regression models are shown as violin plots. Points are species used in this study, open and closed circles indicate *p* ≥ 0.05 and *p* < 0.05, respectively. LA, lamina area; LT, lamina thickness; SLA, specific lamina area; Lchl, lamina chlorophyll content; LDMC, lamina dry matter content; PD, petiole diameter; PL, petiole length; SPL, specific petiole length; PDMC, petiole dry matter content; LMR, lamina matter ratio. (Online version in colour.)

Leaf traits varied strongly with increased seedling height, especially for lamina traits (figure 2). In general, seedling leaves became larger and tended towards the resource-conservative strategy with increased height (figure 2). Taller seedlings exhibited larger LA, thicker LT and petiole diameter, and longer petiole length, as well as lower SLA, specific petiole length, lamina chlorophyll content, and higher lamina, and petiole dry matter content (figure 2). Most species showed an increased lamina matter ratio with increased height, which represented more leaf biomass was allocated to lamina rather than petiole (figure 2). Especially, only one species (*Maackia amurensis*) had more resource-acquisitive laminas with increased height (figure 2). Petiole traits and the lamina matter ratio showed a larger variation among species, for example, *Eleutherococcus senticosus* and *Euonymus verrucosus* had shorter petioles with increased height and *Fraxinus mandschurica* showed lower lamina matter ratio with increased height.

Compared to height, leaf traits of a few species were influenced by light and elevation. While no obvious change was found for LA, leaves tended to be thicker and showed the resource-conservative strategy with increased light availability (electronic supplementary material, figure S4). Petiole traits did not exhibit strong responses to light availability except for four species that showed longer petioles in the high-light environments (electronic supplementary material, figure S4). Among 10 leaf traits, only lamina economics spectrum traits showed similar responses among species to the elevational gradient where higher SLA and Lchl, and lower LDMC were found in the high elevation environments (electronic supplementary material, figure S5).

### (b) Leaf traits influence seedling survival and growth

**Figure 3.**
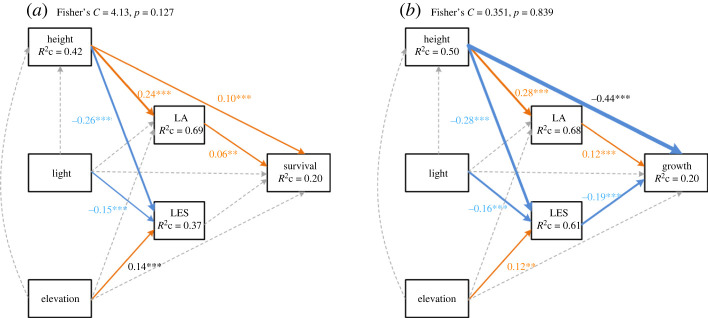
Structural equation models of seedling survival (*a*) and growth (*b*). LA, lamina area; LES, lamina economics spectrum. A higher LES value represents a more resource-acquisitive strategy. *R*^2^_c_ is the conditional *R*^2^ in the mixed model. Grey dashed lines indicate non-significant relationships (*p* ≥ 0.05), solid brown lines indicate significant positive relationships (*p* < 0.05) and solid blue lines indicate significant negative relationships. Line width indicates correlation strength. (Online version in colour.)

Of all 2632 seedlings, 988 (60.1%) seedlings died from 2018 to 2020. Similar to results from multiple regression models, structural equation modelling showed that height was positively correlated with LA and negatively correlated with LES; light availability was negatively correlated with LES and elevation showed a positive correlation (figure 3). Height appeared to be positively correlated with seedling survival directly and indirectly with increased LA (figure 3*a*). Light availability and elevation did not influence seedling height and survival (figure 3*a*).

For all 998 seedlings that survived in 2020 with growth data, the relative growth rate of height was 0.099 ± 0.087 (mean ± s.d.). While height showed a strong negative effect on seedling growth directly in the structural equation modelling, the increased LA and decreased LES with increased seedling height were correlated with faster seedling growth (figure 3*b*). Light and elevation did not show a direct effect on seedling growth, but show an indirect effect by mediating leaf trait variation (figure 3*b*): increasing light availability decreased LES to indirectly increase seedling growth and higher elevation would increase LES to indirectly decrease seedling growth.

### (c) Relationships between species-specific variation in traits, survival and growth

**Figure 4.**
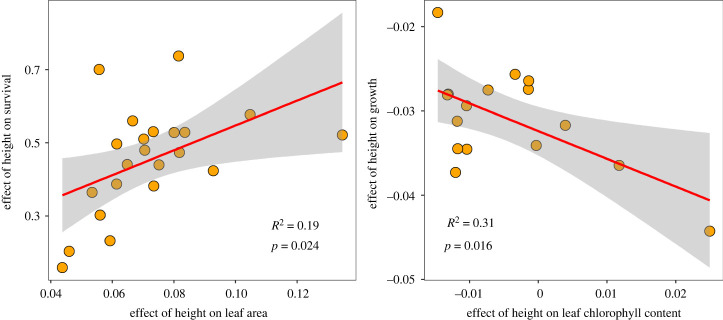
Relationships between species-specific coefficients of height in trait models and those in survival and growth models. Each point represents one species. (Online version in colour.)

We found that the species-specific variation in seedling traits could explain species-specific variation in seedling survival and growth along with height and environmental gradients, which supported our prediction shown in figure 1*c* (table 1 and figure 4). Especially, species with a faster-increased LA with increased height showed a faster-increased survival probability (*p* = 0.024, figure 4). Similarly, species with a faster-decreased Lchl with increased height showed a slower-decreased growth rate (*p* = 0.016, figure 4). In growth models, species-specific variation in LA and LT also showed marginally significant effects to drive the species-specific variation in growth rate along with the light gradient (table 1).

**Table 1.** Relationships between species-specific coefficients of height, light, and elevation in trait models (equation (2.7)) and those in survival (equation (2.5)) and growth (equation (2.6)) models. Italicized font indicates *p* < 0.1, bold font indicates *p* < 0.05.

trait model	variable	survival model			growth model		
		slope	*p*-value	*R*^2^	slope	*p*-value	*R*^2^
lamina area	height	**3.39**	**0.024**	**0.190**	0.08	0.241	0.032
	light	−6.72	0.680	−0.041	*2.68*	*0.074*	*0.154*
	elevation	−6.69	0.114	0.076	0.14	0.700	−0.060
lamina thickness	height	−4.26	0.629	−0.037	0.16	0.703	−0.060
	light	−1.48	0.531	−0.029	*0.49*	*0.055*	*0.185*
	elevation	1.11	0.810	−0.047	0.02	0.955	−0.071
specific lamina area	height	−2.14	0.562	−0.032	−0.05	0.762	−0.064
	light	0.30	0.945	−0.050	−0.51	0.209	0.047
	elevation	5.28	0.275	0.012	0.28	0.633	−0.054
lamina chlorophyll content	height	−5.50	0.115	0.075	**−0.33**	**0.016**	**0.305**
	light	2.62	0.224	0.027	3.63	0.327	0.002
	elevation	1.93	0.681	−0.041	0.15	0.648	−0.055
lamina dry matter content	height	−6.75	0.373	−0.008	0.12	0.722	−0.061
	light	2.25	0.643	−0.038	0.05	0.926	−0.071
	elevation	−7.84	0.466	−0.022	−0.38	0.636	−0.054

## 4. Discussion

Using an individual-level dataset in a temperate forest, we examined the role of plant functional traits on the seedling size–demography (i.e. survival and growth) and environment–demography relationships. We found many species showed large trait variation with increased seedling height, but the environmental influences on leaf traits were relatively weak. Seedling survival and growth were strongly related to height directly and indirectly to the height-related leaf trait variation. We did not find the direct effects of environmental factors on seedling survival and growth, instead, leaf trait plasticity along our observed environmental gradients determined seedling growth. Finally, species-specific variation in traits could explain species-specific variation in survival and growth along with the plant size gradient. Our results suggest that plant functional traits are important mediators on the usually observed plant size–demography and environment–demography relationships in previous studies.

### (a) How do leaf traits vary with increased seedling height, light availability and elevation?

Using an individual-level trait dataset for multiple co-occurring species in a temperate forest, we found that leaf traits varied along with height and environmental gradients. Although the trait variation of some species was not significant, the trends in trait variation were similar among species. First, a whole leaf was larger when a seedling was taller, including larger lamina area and thickness, as well as petiole diameter and length, suggesting a coupled relationship between plant size and the size of these organs [28,35]. Second, the increased conservative leaf traits for larger plants in our study were also observed in other studies [36]. However, most previous studies focused on plants at later life stages. For example, Damián *et al*. [37] and Dayrell *et al*. [38] found adult trees had larger leaf areas and more conservative traits than their juveniles. Park *et al*. [22] found increased leaf mass per area (1/SLA) with increased DBH for trees with DBH > 1 cm. However, Martin & Thomas [21] found leaf area showed a unimodal or decreased relationship between plant DBH and leaf area. This evidence suggests that trait variation with plant size may be varied among species and between life stages.

More conservative strategies for leaves in the high-light conditions were also found in other studies [39,40]. While larger leaf area per dry matter might increase light interception in the low-light conditions, larger leaf matter with increased photosynthetic biomass per area will increase photosynthetic capacity in the high-light conditions [39]. Surprisingly, we found SLA and lamina chlorophyll content for most species increased with elevation. These trends of trait variation were opposite to what previous studies have found [17,41], namely that SLA is higher at low elevations. These results indicate that trait variation patterns with environmental factors may be different between seedlings and large trees.

### (b) How do leaf traits mediate seedling survival and growth along with height and environmental gradients?

Seedling size–demography and environment–demography relationships are well understood by previous studies [1,4,5]; however, the role of functional traits underlying these patterns is usually overlooked. We found that environmental factors did not influence seedling demography directly, which did not support the environmental filtering hypothesis [16], where researchers often assume a direct link between environmental conditions and fitness, at least for the environmental factors we measured in this study. Instead of the usually assumed abiotic stresses, our results supported that the environment-driven trait variation influenced seedling demographic rates (trait plasticity hypothesis). Environment-driven trait variation can be contributed by species turnover and intraspecific trait variation, which both influence community dynamics along environmental gradients. However, environmental filtering might be found if we included other environmental factors. Excluding seedlings with height < 10 cm also likely weaken our ability to find the effects of environments on seedling survival and growth because non-random mortality of plants is usually very high at the young life stage [42]. Therefore, we do not suggest that environmental filtering is not important in community assembly, instead, we want to emphasize that the environment-driven trait variation, a usually overlooked process, is also important to determine community dynamics along environmental gradients. These results suggest that both environment-driven trait variation and environmental filtering processes potentially contribute to community dynamics change along environmental gradients. Further, as Cadotte & Tucker [16] argue, environmental variation might drive competitive differences and this competition asymmetry–environment relationship should be mediated by trait differences. This would be a fruitful future avenue for research.

We found that only LA showed a positive effect on seedling survival, which was consistent with our previous study [43]. A larger leaf area could determine seedling light interception efficiency [44]. Most previous evidence suggests that there is a survival-growth trade-off across species [45–47], where fast-growing species show low survival rates. However, we found that increased acquisitive traits (e.g. higher SLA) decreased seedling growth rates, which did not support this hypothesis. Many reasons likely contributed to this result. First, the individual-level trait–demography relationship might be different from that using species-level trait values. For example, Umaña *et al*. [32] used individual-level data and found seedlings with more acquisitive traits had lower growth rates, which was not found in their species-level trait data. Second, the trait–demography relationship may be different between seedlings and trees. Compared to trees, shade tolerance is more important for seedlings [48]. For temperate deciduous species, seedlings with lower SLA are more shade-tolerate and will have a high photosynthetic rate under limited light conditions and thus have a high growth rate [49,50]. Finally, after controlling the effects of leaf trait variation in our structural equation models, height still showed a very strong and direct effect on seedling survival and growth. This might be because other height-related functional traits (e.g. defence traits or traits from other organs, [51]) potentially influenced seedling survival and growth. Also, higher light availability for taller seedlings might increase survival probability.

### (c) Species-specific trait variation determines how their demographic rates change along with height and environmental gradients

According to our results above, seedlings usually showed species-specific variation in traits and demography along with the height and environmental gradients, and we found height-related trait variation was correlated with the height-related survival and growth variation across species. Consistent with the results from structural equation modelling, species-specific variation in LA with height could influence species-specific variation in survival, species-specific variation in leaf economics spectrum traits (i.e. lamina chlorophyll content) with height determined species-specific variation in growth rates. Similarly, marginally significant results were found for LA and lamina thickness along with the light gradient. These relationships strengthened our evidence that functional traits are important mediators to influence both the whole community dynamics and species-specific dynamics across abiotic and biotic gradients [31,52]. These results are attractive but also this line of research needs more work, for example, to understand why species show different trait variations along with plant size and environmental gradients. Finally, our study suggests that it is useful for us to consider the role of functional traits when evaluating the size–demography and environment–demography relationships.

## 5. Conclusion

Functional traits are always expected to influence plant demographic rates; however, scarce evidence exists for how functional traits mediate the plant ontogeny/size–demography and environment–demography relationships (but see [31,52]), especially at the seedling stage which experiences most of a population's mortality [1]. Here we used an individual-level trait dataset combined with seedling survival and growth dynamics, to explore how leaf traits influenced survival and growth under the changing ontogenetic and environmental contexts in a natural forest. We found seedling leaf traits varied strongly along with height than environmental gradients, which showed larger leaf size and a more conservative strategy for taller seedlings. Seedling height showed direct effects on survival and growth and indirect effects by mediating trait variation. Finally, species-specific trait variation along with the height and environmental gradients could explain why different seedling species showed different demographic dynamics along these gradients. Our study highlights functional traits play a more important role than ever thought to influence community dynamics along biotic and abiotic gradients.

## Data Availability

Data available from the Dryad Digital Repository: https://doi.org/10.5061/dryad.7sqv9s4vw [53]. The data are provided in the electronic supplementary material [54].
